# Blown Composite Films of Low-Density/Linear-Low-Density Polyethylene and Silica Aerogel for Transparent Heat Retention Films and Influence of Silica Aerogel on Biaxial Properties

**DOI:** 10.3390/ma15155314

**Published:** 2022-08-02

**Authors:** Seong Baek Yang, Jungeon Lee, Sabina Yeasmin, Jae Min Park, Myung Dong Han, Dong-Jun Kwon, Jeong Hyun Yeum

**Affiliations:** 1 Department of Biofibers and Biomaterials Science, Kyungpook National University, Daegu 41566, Korea; ysb@knu.ac.kr (S.B.Y.); dlwjddjs2@gmail.com (J.L.); yeasminsabina44@yahoo.com (S.Y.); woawoa9025@naver.com (J.M.P.); 2 Hans Intech Co., Ltd., Daegu 41243, Korea; mdhan@hanschemtek.com; 3 Department of Materials Engineering and Convergence Technology, Research Institute for Green Energy Convergence Technology, Gyeongsang National University, Jinju 52828, Korea

**Keywords:** blends, composites, blown film extrusion, silica aerogel, biaxial properties, morphology, thermal properties, mechanical properties

## Abstract

Blown films based on low-density polyethylene (LDPE)/linear low-density polyethylene (LLDPE) and silica aerogel (SA; 0, 0.5, 1, and 1.5 wt.%) were obtained at the pilot scale. Good particle dispersion and distribution were achieved without thermo oxidative degradation. The effects of different SA contents (0.5–1.5 wt.%) were studied to prepare transparent-heat-retention LDPE/LLDPE films with improved material properties, while maintaining the optical performance. The optical characteristics of the composite films were analyzed using methods such as ultraviolet–visible spectroscopy and electron microscopy. Their mechanical characteristics were examined along the machine and transverse directions (MD and TD, respectively). The MD film performance was better, and the 0.5% composition exhibited the highest stress at break. The crystallization kinetics of the LDPE/LLDPE blends and their composites containing different SA loadings were investigated using differential scanning calorimetry, which revealed that the crystallinity of LDPE/LLDPE was increased by 0.5 wt.% of well-dispersed SA acting as a nucleating agent and decreased by agglomerated SA (1–1.5 wt.%). The LDPE/LLDPE/SA (0.5–1.5 wt.%) films exhibited improved infrared retention without compromising the visible light transmission, proving the potential of this method for producing next-generation heat retention films. Moreover, these films were biaxially drawn at 13.72 MPa, and the introduction of SA resulted in lower draw ratios in both the MD and TD. Most of the results were explained in terms of changes in the biaxial crystallization caused by the process or the influence of particles on the process after a systematic experimental investigation. The issues were strongly related to the development of blown nanocomposites films as materials for the packaging industry.

## 1. Introduction

Polymer blending is defined as a process in which at least two polymers are blended to produce a new material with different physical characteristics. This is mainly performed to improve and increase certain characteristics such as the thermal barrier [1]. Blends of linear low-density polyethylene (LLDPE) and low-density polyethylene (LDPE) are of considerable importance in industrial applications. Their good processability and excellent mechanical properties make LDPE/LLDPE films suitable for packaging applications. LLDPE is added to LDPE because of its superior mechanical characteristics such as higher tensile strength, impact properties, and elongation at break. The LDPE/LLDPE films are characterized by low haze and better bubble stability. Furthermore, manufacturers can use conventional LDPE film-blowing devices to blend LLDPE with LDPE without modification [2]. Despite the benefits of LDPE/LLDPE blends in film applications, blend miscibility, and the miscibility of LDPE/LLDPE blends have certain effects on their properties, and few studies have been conducted on the miscibility of LDPE/LLDPE blends [3,4,5,6]. Most researchers have reported that LLDPE/LDPE blends are miscible at low LDPE contents and show immiscibility at higher LDPE [7]. In this study, the LDPE concentrations were kept low, and the differential scanning calorimetry (DSC) thermograms exhibited two overlapping peaks, which may be due to the phase separation of the LDPE and LLDPE components in the blended film after crystallization [8]. Although LDPE and LLDPE resins are the most versatile polymers, their applications are restricted by drawbacks such as low strength, stiffness, and poor heat resistance [9]. To solve these problems and prepare materials with improved properties, the preparation of polyethylene (PE) nanocomposites with different inorganic nanofillers has been reported [10,11].

Currently, energy cost and availability are important concerns, and heat energy thermal insulation is an efficient strategy to address these issues. Low thermal conductivity is considered to be an essential feature of materials that can be reinforced by incorporating fillers into the main matrix [12]. Nanoporous networks of aerogels filled with gas (above 90% of aerogels are composed of air) show excellent characteristics including high specific surface area (500–1200 m^2^/g), low thermal conductivity (0.013–0.04 W/m K), low dielectric constant (1.1–2), low density (0.003–0.1 g/cm^3^), high optical transmission in the visible range (90%), and high insulating ability. These properties make them good candidates as insulators in different applications [13]. Low thermal conductivity is one of the major characteristics of silica aerogels (SAs), making them applicable in the field of insulation. However, SAs have poor mechanical properties, which restrict their application. Therefore, SA-based composites are generally used [14,15].

Blown film extrusion is the main processing method for producing a biaxial melt-drawn film. This method requires the use of air pressure for initiating a transverse direction (TD) draw, in addition to a higher haul-off roll speed for delivering a machine direction (MD) draw. Billions of pounds of polymer are processed annually by using this technique. Blown film extrusion is used to produce agricultural and construction films, industrial films and bags, stretch films, polyvinyl chloride cling films, liners, high barriers, and small tube systems [16,17].

The purpose of this study was to improve both the thermal and mechanical properties of the LDPE/LLDPE blend films; hence, LDPE/LLDPE/SA composites were prepared using a twin-screw extruder to prepare an LDPE/LLDPE/SA (0–1.5 wt.%) masterbatch, and then an LDPE/LLDPE/SA (0–1.5 wt.%) film was prepared using the blown method [18,19]. The effects of various SA contents on the morphology, draw ratio, and mechanical and thermal characteristics of the prepared composite films were studied. Our proposed blown film extrusion of composites based on LDPE/LLDPE and SA could widen their application as thermal insulating films in the packaging field while preserving the biaxial film properties initiated by the processing method or the impact of the particles on the processing method.

## 2. Experimental Section

### 2.1. Materials

Polymer samples of LDPE and LLDPE were purchased from Equate and Seongji Industrial Co., Ltd. (Apryang-myeon, Gyeongsan-si, Korea), and the material characteristics of both polymers are listed in Table 1 (Part a). The SA powder was provided by EM-POWER Co., Ltd. (Asan-si, Chungnam, Korea), and its properties are listed in Table 1 (Part b).

### 2.2. Preparation of LDPE/LLDPE/SA (0–1.5 wt.%) Extruded Blown Composite Film

**Figure 1:**
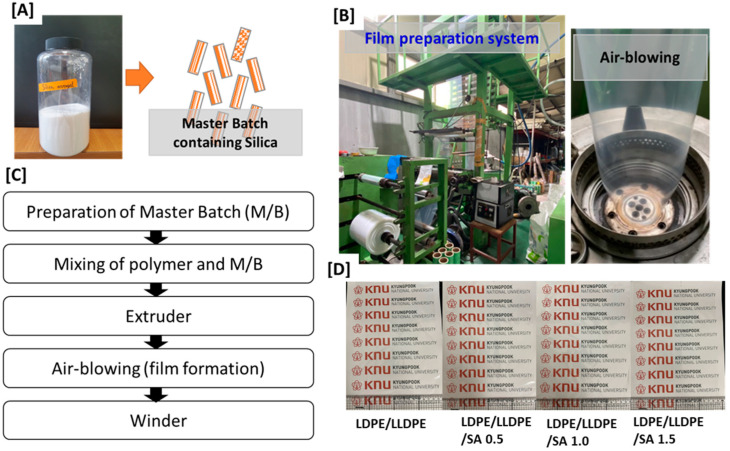
A demonstration of the experiment. (**A**) SA powder and masterbatch containing SA; (**B**) film preparation system at the pilot scale; (**C**) diagram showing the preparation of the air blown film; and (**D**) a macro photograph of the prepared films.

Figure 1 displays the preparation method of the LDPE/LLDPE/SA masterbatch and film. Before the preparation of the LDPE/LLDPE masterbatch (30%/70%) with various SA contents (0–1.5 wt.%), the feed rate was set by calculating the weight of the LDPE, LLDPE, and SA exiting through the feeder. The screw rate was fixed at 480 rpm, at 150–160 °C, and extruded by mixing LDPE, LLDPE, and SA (0–1.5 wt.%) through the feeder. LDPE/LLDPE/SA was fed into the twin-screw extruder via the hopper and discharged through the feeder at a fixed rate. The extrudate was appropriately cooled through the water-cooling zone and cut using a pelletizer to obtain a masterbatch chip. The prepared masterbatch of LDPE/LLDPE/SA with various SA contents (0–1.5 wt.%) was fed to a blown film maker with LDPE/LLDPE in a constant ratio to prepare the LDPE/LLDPE/SA composite film. For the air-blown pure-blend LDPE/LLDPE film, any further addition of LDPE/LLDPE to the LDPE/LDPE masterbatch chip was not necessary.

### 2.3. Instrumental Analysis

The dispersion states and morphologies of the LDPE/LLDPE/SA composites with various SA contents (0–1.5 wt.%) were tested by applying scanning electron microscopy (SEM, SU8220, Hitachi, Japan) at an accelerating voltage of 10.0 kV. A gold coating was applied to each sample before the analysis. The crystallization behavior of the LDPE/LLDPE/SA (0–1.5 wt.%) composites were studied by performing X-ray diffraction (XRD, D/Max–2500, Rigaku, Tokyo, Japan) using Cu-Kα radiation under operational conditions of 40 kV with 2θ in the range of 2°–40°, having a step interval of 0.02°. Differential scanning calorimetry (DSC) (Q 2000, TA Instruments, New Castle, DE, USA) was performed to analyze the thermal characteristics and crystallinity of the LDPE/LLDPE/SA (0–1.5 wt.%) composite films. First, all of the samples were exposed to a temperature from 30 °C to 300 °C at a heating rate of 10 °C min^−1^, then kept for 10 min at 300 °C to remove the thermal history, and subsequently cooled down to 30 °C at a cooling rate of 10 °C min^−1^. A second heating method was performed at 300 °C at a similar scanning rate. The degree of crystallinity (X_c_) was calculated from the peak area of the DSC thermograms [17]. The mechanical properties were evaluated using an Instron 5567 material testing system at 25 °C, as per the ASTM D638-96 type II requirements [18]. All data were estimated based on the average of three sample measurements. The light transmittance (T%) of the film was measured by employing ultraviolet–visible (UV–Vis) spectroscopy (K Lab Co., Ltd., Optizen O 2120UV, Daejeon, Korea) in the 200–800 nm wavelength range. The infrared thermal images were captured by a FLIR system AB (Täby, Sweden) infrared thermal imager.

## 3. Results and Discussion

**Figure 2:**
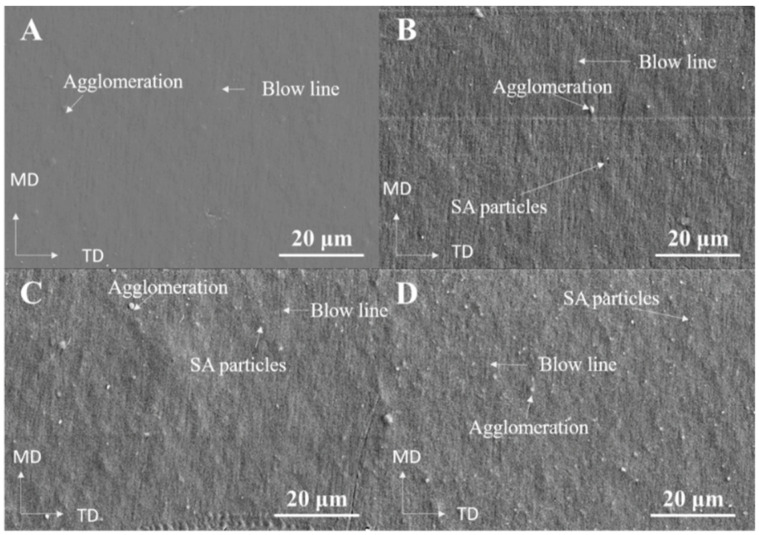
The field-emission scanning electron microscopy (FE-SEM) images of the LDPE/LLDPE blend polymer film with various SA contents: (**A**) 0 wt.%, (**B**) 0.5 wt.%, (**C**) 1 wt.%, and (**D**) 1.5 wt.% (MD = machine direction and TD = transverse direction).

**Figure 3:**
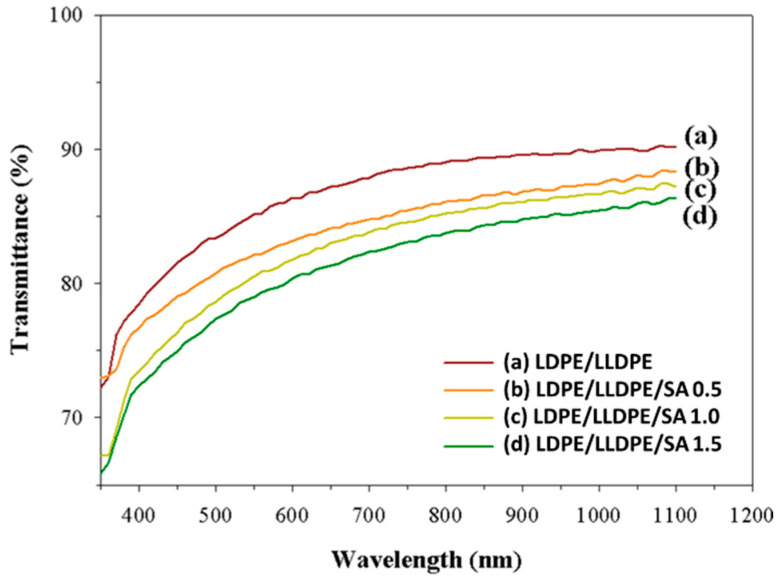
The UV–Vis spectra of the LDPE/LLDPE blend polymer film with various SA contents: (a) 0 wt.%, (b) 0.5 wt.%, (c) 1 wt.%, and (d) 1.5 wt.%.

Microstructural FE-SEM micrographs of the prepared samples with various SA contents are shown in Figure 2. The FE-SEM images clearly show the three-dimensional structure of SA and the effect of SA content on the surface morphology of the LDPE/LLDPE blend film (Figure 2A–D). These results show that the smoothest surfaces were obtained for the LDPE/LLDPE blend film without SA (Figure 2A), and all blown composite films exhibited a good SA distribution in both MD and TD. These results are very significant when considering the mechanical properties of the composite films. However, spherical particles were observed, and the surface roughness increased due to aggregation as the concentration of SA increased (Figure 2B–D) [19]. The optical properties of the LDPE/LLDPE blend films containing different SA contents were determined by measuring the transmittance in the 200–1100 nm range, and the results are presented in Figure 3. The transparency of all of the polymer composite films (0–1.5 wt.%) was good in the visible light range (380–700 nm), in spite of a slight decrease in the transparency with an increase in the content of SA. SA may scatter and absorb ultraviolet light, indicating that it has a UV-blocking function. Photographs of the blended films with different SA contents are exhibited in Figure 1D. All of them showed good visible light transmittance capacity. Similar results were obtained for the LDPE/silica nanocomposite films, showing that various silica contents (0.5–1.5 wt.%) had no significant influence on the transmittance [20].

**Figure 4:**
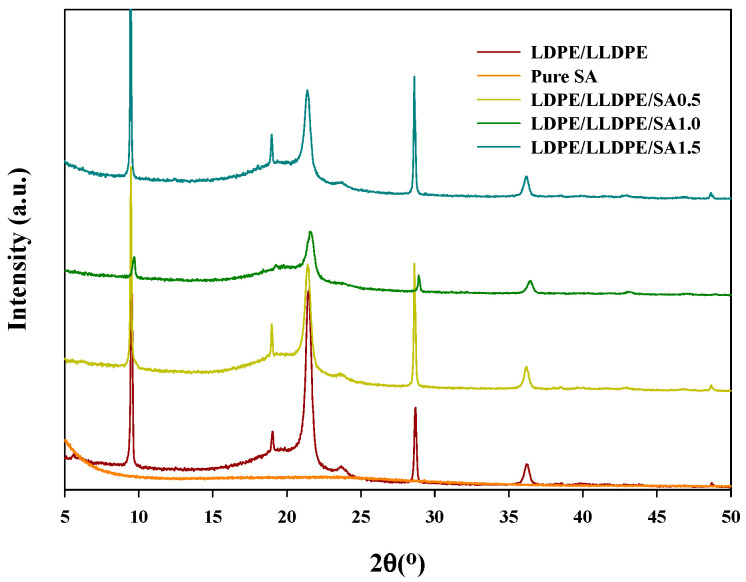
The XRD data of the pure SA and LDPE/LLDPE blend polymer film with various SA contents (0–1.5 wt.%).

Some polymers remain in the crystalline state because their molecular chains can be stretched and narrowly arranged in parallel. PE is an orthorhombic crystalline polymer. The crystallization characteristics have an important effect on the physical properties, melting point, and mechanical strength of the polymers. Therefore, it is crucial to investigate the changes in the crystallinity of PE [21]. The XRD patterns of the LDPE/LLDPE composite films with various SA contents are shown in Figure 4. From these scans, it can be observed that the two major peaks of PE were mainly presented in the 2θ range of 10° to 30°.

The XRD peak at 21.6° was ascribed to the 110 reflections of PE [22]. No obvious peaks of SA appeared in the XRD pattern of the LDPE/LLDPE/SA composite film, indicating that the SA was fully exfoliated. Silica is an amorphous solid, and the amorphous nature of silica was confirmed by a broad peak (2θ = 22°, (101)) in the XRD pattern of pure silica [23]. A large peak at 22° also occurred in our investigation for the pure SA. When a small amount (0.5–1.5 wt.%) of SA was added to the LDPE/LLDPE blend, the absence of the main peaks of SA in the spectra of the composite films may have been caused by the masking effect of the LDPE/LLDPE blend matrix due to the small content of SA [21]. However, it clearly had an impact on the peak intensity of PE. Additionally, after the addition of SA (0.5–1.5 wt.%), a modest shift in the peak location of PE, particularly in the range of 20° to 22°, was seen. These findings can be quite convincingly explained by the various material properties that are brought about by adding SA (0.5–1.5 wt.%), either as a consequence of the process being affected by particles or as a result of the variation in SA dispersion in the extrusion blown LDPE/LLDPE blend matrix with an increase in the SA concentration. The corresponding SEM images of the samples support the XRD measurements [22].

**Figure 5:**
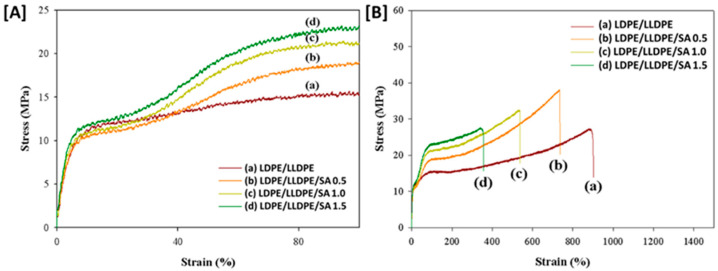
The stress–strain curve for the LDPE/LLDPE blend polymer films with various SA contents: (a) 0 wt.%, (b) 0.5 wt.%, (c) 1 wt.%, and (d) 1.5 wt.%. (**A**) from 0 to 90% and (**B**) from 0 to 1500%. The samples were cut along the MD.

Figure 5 displays the stress–strain curves for the pure LDPE/LLDPE 70/30 blend and the LDPE/LLDPE/SA blown film with various SA contents (0–1.5 wt.%) in the MD, and the tensile properties of the studied films are summarized in Table 2. According to Figure 5B, the stress at break value of the pure blend film increased after adding the SA content (0.5–1.5 wt.%) and the composite with 0.5 wt.% of SA loading showed the highest stress at break (37.96 MPa). In this case, the enhancement of the tensile properties can be clarified by considering the possible influence of SA on the molecular orientation during the extrusion blowing along the MD as well as the good matrix–particle adhesion [24], consequently, promoting the increase in the tensile strength of the composite film with the addition of SA particles (0.5–1.5 wt.%). However, if the SA amount exceeded 0.5%, the stress at break decreased gradually from 37.96 to 27.72 MPa. In contrast, the stress–strain curves illustrated that the strain at break values of the LDPE/LLDPE blend film gradually decreased from 882.18% to 349.01% with the incorporation of SA (0.5–1.5 wt.%). The SA particles may be stuck inside the entanglements, thus resulting in a restriction in the polymer’s total chain mobility [20].

**Figure 6:**
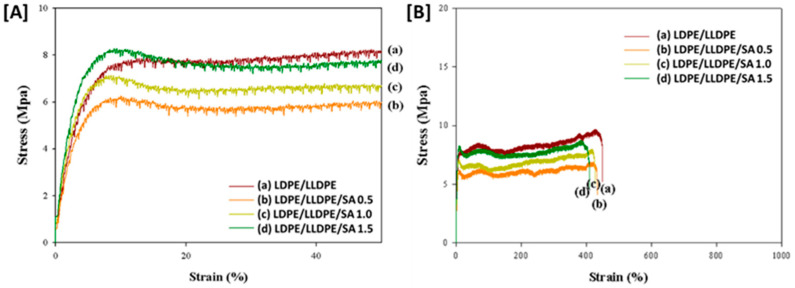
The stress–strain curve for the LDPE/LLDPE blend polymer film with various SA contents: (a) 0 wt.%, (b) 0.5 wt.%, (c) 1 wt.%, and (d) 1.5 wt.%. (**A**) from 0 to 90% and (**B**) from 0 to 1000%. The samples were cut along the TD.

The gradual decrease in mechanical properties above a 0.5 wt.% SA loading may deteriorate the dispersion in the LDPE/LLDPE blend solution, as mentioned earlier [25]. In addition, agglomerated SA particles exhibit poor tensile properties. In this study, the SA particles were well-dispersed at lower loading (0.5 wt.%), showing excellent reinforcing efficiency. Moreover, as shown in Figure 5A, the stress increased gradually with filler loading (0.5–1.5 wt.%) within a lower (90%) strain. This behavior is likely to be related to the stiff layers of silicate with a high aspect ratio, which produce a high degree of interaction and appropriate interfacial adhesion properties. Moreover, this tendency restricts the free movement of the polymer chains, increasing the tensile strength value [26]. However, when the strain values increased, a strain hardening mechanism developed, which may be a result of an orientated crystalline structure of polymer in both MD (Figure 5B) and TD (Figure 6B) [25]. Nanocomposites containing various SA concentrations (0.5–1.5%) showed different strain hardening mechanisms than the pure blend film, as was expected by considering the influence of SA dispersion on the polymer chain orientation along the blow direction during the film-blowing [27], which was also demonstrated by the yield point at low strain values (Figure 5A and Figure 6A) and that, with an increase in SA content (0.5–1.5%), the yield stress and yield strain of the blown film, as shown in Table 2 and Table 3, increased from 6.34–7.13%, and 9.92–11.49 MPa, respectively, for MD and by 7.77–8.28%, 6.11–8.25 MPa, respectively, for TD (i.e., the mechanical strength and flexibility increased as the SA content increased). The crystallinity of the blown film (Table 4) was also influenced by the effect of SA on the molecular chain, as the molecular orientation decreased the polymer fractional free volume and molecular flexibility, and induced crystallinity [28].

Because of the effect of the film-blowing ratio and traction ratio in the film-blowing procedure, the mechanical behavior of the film in different directions was different, and the MD film performance (Figure 5) was often higher than the TD film performance (Figure 6) [29]. In the composite films, the nanoparticles spread in a preferential position during blown film extrusion, which could result in changes in the mechanical properties [30]. To determine the influence of processing and particle existence on the mechanical behavior of the blown films, a stress–strain test was also performed in the TD, as presented in Figure 6, and the mechanical properties are summarized in Table 3. The mechanical properties of the LDPE/LLDPE/SA (0.5–1.5 wt.%) films in both directions, MD and TD, had a different behavior than those of the pure blend LDPE/LLDPE film. Unlike the MD samples (27.73–37.96 MPa) presented in Figure 5, the samples in the TD showed lower stress at break (6.31–8.05 MPa) compared to the unfilled blend matrices; this can be related to the low ductility of the composite films compared to the unfilled blend matrix, resulting in early failure of the samples during the test [31]. The difference in molecular chain orientation and the effect of SA particle dispersion on the molecular chain orientation along the blow direction (MD) may also be responsible for this difference between the MD and TD film samples. This difference may allow for a differential increase in the composite film stiffness (Table 2 and Table 3) [32]. Moreover, according to Figure 5A and Figure 6A, the stress–strain curves of the LDPE/LLDPE/SA (0.5–1.5 wt.%) composite films in both directions showed the same characteristic ductile deformation behavior of semicrystalline polymers [33] and the reinforcing effect of SA particles.

**Figure 7:**
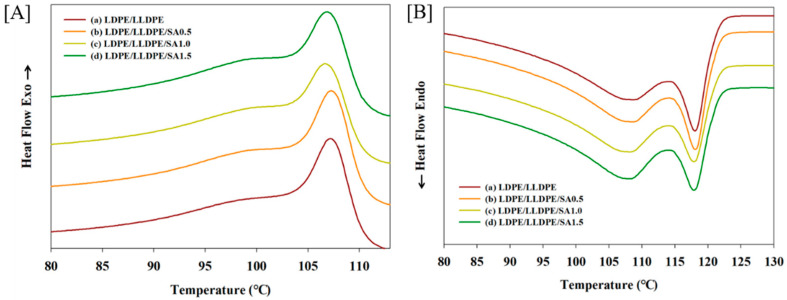
The DSC cooling curves (**A**) and heating curves (**B**) for the LDPE/LLDPE blend film with various SA contents: (a) 0 wt.% (b) 0.5 wt.%, (c) 1 wt.%, and (d) 1.5 wt.%.

The crystallization behaviors of the LDPE/LLDPE/SA composite films with various SA contents (0–1.5 wt.%) were studied by DSC, and the resulting DSC cooling and heating curves are shown in Figure 7.

The experimental results in terms of the melting temperature (T_m_), crystallization temperature (T_c_), crystallinity (X_c_), and heat of crystallization (ΔH_c_) are listed in Table 4. The crystallization temperature, enthalpy of crystallization, and X_c_ were obtained from the cooling cycle (exothermal peak) (Figure 7A). The second heating run for the neat LDPE/LLDPE blend composite film and those with various loadings of SA (0.5–1.5 wt.%) are shown in Figure 7B and the data are listed in Table 4. The T_m_ was calculated based on the second heating cycle (Figure 7B) and the two melting temperatures (T_m1_ and T_m2_) for all of the studied films are listed in Table 4, corresponding to the first and second endotherms, respectively.

According to Table 4, the addition of SA, irrespective of its content, widened the crystallization window (increasing ΔT = T_onset_–T_peak_) and delayed the crystallization progression. This is because SA increases the viscosity of the LDPE/LLDPE matrix, hampers LDPE/LLDPE chain movements, and slows down crystallization development. The composite with 0.5 wt.% SA loading yielded the highest crystallinity (X_c_%, 33.1%). However, if the SA content exceeded 0.5%, the crystallinity decreased, and the heat enthalpy of the crystallization (ΔH_c_) results showed the same tendency (Table 4). This result suggests that at higher SA contents (1–1.5 wt.%), it did not act as a nucleating agent but hampered polymer chain movements by absorbing polymer segments on its surface. Similar findings were reported in a previous study [34]. As expected, the DSC endotherm of the binary blend LDPE/LLDPE sample showed two distinct major peaks (T_m1_, T_m2_) (Figure 7B). A similar feature was shown in the endotherms of the LDPE/LLDPE samples reported earlier [8]. Furthermore, the effect of SA content on the LDPE/LLDPE melting point was carefully observed. As the amount of SA increased in the LDPE/LLDPE blend, the T_m1_ and T_m2_ values decreased, and its intensity changed, which can be attributed to the effect of the filler as it influences the thermal motion of the polymer [35]. It should be mentioned that the MD film was used for the crystallization process and the crystallization curves should not only reflect the general filler effect [36] of SA particles on LDPE/LLDPE crystallization, but also the effect of SA particle dispersion on the molecular chain orientation along the blow direction (MD), as molecular orientation significantly affects the molecular flexibility, which induces the crystallinity [28]. Thus, the variation in the crystalline peak (107.21–106.91 °C) for the extrusion blown LDPE/LLDPE/SA (0–1.5%) system may arise from the strong effect of blown extrusion on the crystal degree orientation [37] and SA aggregates with the increase in the SA concentration as observed through SEM (Figure 2A–D). Additionally, the possible influence of the embedded SA particles on the molecular orientation along the flow direction is also reflected in the mechanical behavior of the film in different directions (Figure 5 and Figure 6) [22].

**Figure 8:**
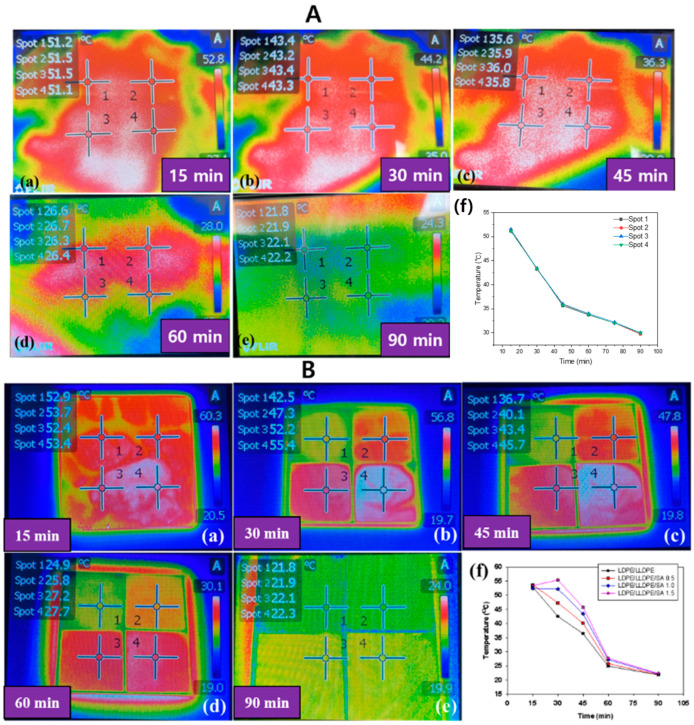
The infrared thermal images and temperature-time curve of (**A**) only the hot plate and (**B**) the prepared LDPE/LLDPE/SA (0–1.5%) films obtained by the hot plate. (**a**–**e**) of (**A**,**B**) are measuring time such as (**a**) 15 min, (**b**) 30 min, (**c**) 45 min, (**d**) 60 min, and (**e**) 90 min. (**f**) represented the temperature according to time.

The thermal insulation performance of the LDPE/LLDPE/SA (0–1.5 wt.%) composite films was confirmed by the IR camera and are presented in Figure 8B. The graph in Figure 8B(f) shows the temperature profiles obtained by placing the LDPE/LLDPE/SA (0–1.5 wt.%) composite films on a hot plate and exposing them to heating for 6 s and subsequent cooling, and after 6 s of heating, the hot plate was turned off, and the temperature was collected from the circle-area marked spots of each film sample, as shown by the temperature–time curve in Figure 8B(a–e). The thickness of all of the studied films is summarized in Table 2. The results show that the film surface temperature decreased, and the heat stored in the film was emitted. After 15 min, all of the studied films showed almost the same temperature; however, after 30 min, the pure blend LDPE/LLDPE film showed a faster cooling rate than the LDPE/LLDPE/SA composite film with various SA contents, and the heat retention capacity increased with the addition of SA (0.5–1 wt.%).

A similar observation was found in a previous report, where it was shown that the addition of SA reduced the thermal conductivity by approximately 30% with 1–2 wt.% of SA loading [22]. Thus, the prepared composite LDPE/LLDPE/SA (0.5–1.5 wt.%) film had a lower thermal conductivity than the neat LDPE/LLDPE blend film, and was suitable for application as a thermally insulating material. A control experiment was also conducted simultaneously (Figure 8A(a–f)), and in contrast to the composite films, the temperature was measured from the circle-area marked spots of only the hot plate surface without any films. The results showed a similar time–temperature relationship (Figure 8A(f)) and were consistent with the results of all of the examined films (Figure 8B(f)), which showed the same time range (15–90 min) to reach room temperature from the starting temperature (51 °C).

**Figure 9:**
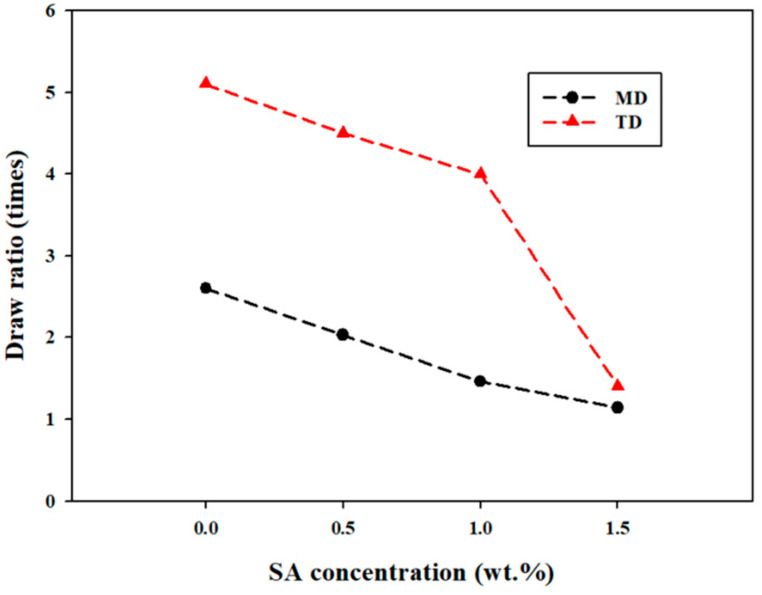
The draw ratio results in the MD and TD of the LDPE/LLDPE blend film as a function of SA content.

Figure 9 depicts the draw ratio at two different directions: MD and TD. As can be observed, a linear decrease in the draw ratio was obtained as a function of the SA content for both directions. However, the main aspect to note in these curves is that the highest maximum draw ratio was achieved for the pure LDPE/LLDPE blend film. Another interesting observation is that all curves showed a similar dependence on the SA content (0–1.5%). This indicates that the decreasing tendency of the draw ratio when increasing the SA content is the result of the direct SA reinforcement effect. As indicated above, the main effect of the addition of SA particles is the decreased drawability of the LDPE/LLDPE matrix. This decreased draw ratio resulted in a restriction in the molecular orientation and chain extension when the SA content increased, which in turn was responsible for the subsequent decrease in the draw ratio in both directions.

Clearly, all of the investigated films, LDPE/LLDPE and the composites incorporating 0.5 wt.%, 1 wt.%, or 1.5 wt.% SA, revealed the same unique relationship between the draw ratio and fillers, revealing that the increase in draw ratio was mainly related to the oriented polymer matrix rather than an additional effect of the filler’s reinforcement. Moreover, the obtained draw ratio for the TD was higher than that for the MD, which may be due to a variation in the orientation of the polymer chain during processing of the blown films.

## 4. Conclusions

Heat-retention LDPE/LLDPE blend films containing highly insulating SA particles (0.5–1.5 wt.%) were prepared successfully by employing blown extrusion at a pilot scale, and the influence of various amounts of SAs on the material properties of composite films was investigated. Macroscopically, the almost homogeneous appearance of the blown films was the result of a good SA dispersion within the LDPE/LLDPE blend, and this was verified by performing SEM, XRD, UV–Vis spectroscopy, and DSC. The SA nucleating capacity was revealed by the increase in the crystallinity degree and crystallization temperature when 0.5 wt.% of SA was incorporated in the composite films. Therefore, the particle dispersion, nucleating character, and strong effect of blown extrusion played a significant role in the introduction of the preferential LDPE/LLDPE crystalline orientation and the properties of the final film. Based on this logic, differences in the tensile behavior of the composite films were observed between the MD and TD. Almost all of the films (LDPE/LLDPE/SA (0–1 wt.%)) showed a higher elongation at break in the MD than in the TD. However, films with 1.5% SA showed almost the same value in both directions. This may be due to the agglomeration at higher SA content, which reduces mobility and elongation. These results confirmed that along with the processing method, the SA particles were also responsible for initiating changes in the biaxial nature of the blown films. Based on the orientation of the dual mechanical characteristics, these composite films can be used as packaging materials in many applications. Moreover, the resulting material showed enhanced infrared retention, and the incorporation of the filler material did not hamper visible light transmission; thus, these films have the potential to be applied in greenhouses with good optical performance.

## Figures and Tables

**Table 1:** (**a**). The material properties of the low-density polyethylene (LDPE)/linear low-density polyethylene samples. (**b**). The technical data of the silica aerogel (SA).

**a.**							
**Name**	**Quantity (g) Used for Blown Film Preparation**	**Grade**	**Melt Flow Index**	**Density**	**Melting Point (°C)**	**Haze (%)**	**Gloss** **(GU)**
LDPE	15,000	LDPE 150E	0.25 g/10 min	0.921 g/cm^3^	96	-	-
LLDPE	35,000	CEFOR 1221P	2.0 g/10 min	0.918 g/cm^3^	116	0.56	151
**b.**							
**Particle Size**	**Pore Diameter**	**BULK DENSITY**	**Surface Chemistry**	**BET** **(Brunauer–Emmett–Teller) Surface Area**		**Porosity**	**Heavy Metal**
20–30 μm	20–30 μm	100 kg/m^2^	Hydrophobic	500 m^2^/g		Less than 90%	N/A

**Table 2:** The mechanical properties of the LLDPE/LDPE/SA (0–1.5%) air blown composite films along the MD.

Specimen	Thickness (µm)	Yield Strain (%)	Yield Stress (MPa)	Stress at Break (MPa)	Strain at Break (%)	Young Modulus (MPa)
LDPE/LLDPE	20 ± 10	8.04	10.75	27.56	882.18	139.55
LDPE/LLDPE/SA 0.5 wt.%	20 ± 11	6.34	9.92	37.96	742.53	152.44
LDPE/LLDPE/SA 1 wt.%	20.8 ± 12	6.60	10.40	32.32	526.72	198.55
LDPE/LLDPE/SA 1.5 wt.%	21.5 ± 13	7.13	11.49	27.73	349.014	222.01

**Table 3:** The mechanical properties of the LLDPE/LDPE/SA (0–1.5 wt.%) air blown composite films along the TD.

Specimen	Thickness (µm)	Yield Strain (%)	Yield Stress (MPa)	Stress at Break (MPa)	Strain at Break (%)	Young Modulus (MPa)
LDPE/LLDPE	20 ± 10	10.42	7.75	8.61	448.202	64.84
LDPE/LLDPE/SA 0.5 wt.%	20 ± 11	7.77	6.11	6.31	430.64	66.13
LDPE/LLDPE/SA 1 wt.%	20.8 ± 12	7.80	7.10	7.88	417.76	88.98
LDPE/LLDPE/SA 1.5 wt.%	21.5 ±13	8.28	8.25	8.05	403.52	94.93

**Table 4:** The DSC analysis data for the LDPE/LLDPE binary blend and LLDPE/LDPE/SA (0–1.5%) air blown composite films.

Sample Type	T_onset_ (°C)	T_endset_ (°C)	T_peak_ (°C)	∆T (°C)	T_m1_ (°C)	T_m2_ (°C)	∆H_c_ (J/g)	X_c_ (%)
LDPE/LLDPE	110.48	63.87	107.21	3.27	108.58	118.05	95.75	32.67
LDPE/LLDPE/SA(0.5)	110.55	63.90	107.25	3.3	108.54	118.02	97.03	33.11
LDPE/LLDPE/SA(1.0)	110.38	63.42	106.74	3.64	108.19	117.86	94.26	32.17
LDPE/LLDPE/SA(1.5)	110.51	63.75	106.91	3.6	108.03	117.86	94.02	32.02

## Data Availability

Not applicable.

## References

[1] Campuzano J., Lopez I. (2020). Study of the effect of dicumyl peroxide on morphological and physical properties of foam injection molded poly (lactic acid)/poly (butylene succinate) blends. Express Polym. Lett..

[2] Zhang Q., Chen W., Zhao H., Ji Y., Meng L., Wang D., Li L. (2020). In-situ tracking polymer crystallization during film blowing by synchrotron radiation X-ray scattering: The critical role of network. Polymer.

[3] Delgadillo-Velázquez O., Hatzikiriakos S., Sentmanat M. (2008). Thermorheological properties of LLDPE/LDPE blends: Effects of production technology of LLDPE. J. Polym. Sci. Part B Polym. Phys..

[4] Fang Y., Carreau P.J., Lafleur P.G. (2005). Thermal and rheological properties of mLLDPE/LDPE blends. Polym. Eng. Sci..

[5] Hussein I.A., Williams M.C. (2004). Rheological study of the influence of branch content on the miscibility of octene m-LLDPE and ZN-LLDPE in LDPE. Polym. Eng. Sci..

[6] Schlund B., Utracki L. (1987). Linear low density polyethylenes and their blends: Part 3. Extensional flow of LLDPE’s. Polym. Eng. Sci..

[7] Al-Attar F. (2018). Thermal, Mechanical and Rheological Properties of Low Density/Linear Low Density Polyethylene Blend for Packing Application. J. Mater. Sci. Chem. Eng..

[8] Monwar M., Yu Y. (2020). Determination of the Composition of LDPE/LLDPE Blends via 13C NMR. Macromolecular Symposia.

[9] Busu W.N.W., Chen R.S., Shahdan D., Yusof M.J.M., Saad M.J., Ahmad S. (2021). Statistical Optimization Using Response Surface Methodology for Enhanced Tensile Strength of Polyethylene/Graphene Nanocomposites. Int. J. Integr. Eng..

[10] Al Sheheri S.Z., Al-Amshany Z.M., Al Sulami Q.A., Tashkandi N.Y., Hussein M.A., El-Shishtawy R.M. (2019). The preparation of carbon nanofillers and their role on the performance of variable polymer nanocomposites. Des. Monomers Polym..

[11] Deng J., Ding Q.M., Li W., Wang J.H., Liu D.M., Zeng X.X., Liu X.Y., Ma L., Deng Y., Su W. (2020). Preparation of nano-silver-containing polyethylene composite film and Ag Ion migration into food-simulants. J. Nanosci. Nanotechnol..

[12] Zolfaghari S., Paydayesh A., Jafari M. (2019). Mechanical and thermal properties of polypropylene/silica aerogel composites. J. Macromol. Sci. Part B.

[13] Chen Y., Klima K., Brouwers H., Yu Q. (2022). Effect of silica aerogel on thermal insulation and acoustic absorption of geopolymer foam composites: The role of aerogel particle size. Compos. Part B Eng..

[14] Tang R., Hong W., Srinivasakannan C., Liu X., Wang X., Duan X. (2022). A novel mesoporous Fe-silica aerogel composite with phenomenal adsorption capacity for malachite green. Sep. Purif. Technol..

[15] Yi Z., Zhang X., Yan L., Huyan X., Zhang T., Liu S., Guo A., Liu J., Hou F. (2022). Super-insulated, flexible, and high resilient mullite fiber reinforced silica aerogel composites by interfacial modification with nanoscale mullite whisker. Compos. Part B Eng..

[16] Ahmed S.S.A.M. (2021). A Sustainable Approach to Reduce Environmental Threats of Oxidative Degradation of Plastic Films. Ph.D. Thesis.

[17] Van de Voorde B., Katalagarianakis A., Huysman S., Toncheva A., Raquez J.-M., Duretek I., Holzer C., Cardon L., Bernaerts K.V., van Hemelrijck D. (2022). Effect of extrusion and fused filament fabrication processing parameters of recycled poly (ethylene terephthalate) on the crystallinity and mechanical properties. Addit. Manuf..

[18] Yeasmin S., Yeum J.H., Yang S.B. (2020). Fabrication and characterization of pullulan-based nanocomposites reinforced with montmorillonite and tempo cellulose nanofibril. Carbohydr. Polym..

[19] Oh S., Shin C., Kwak D., Kim E., Kim J., Bae C., Kim T. (2022). Effect of ionic strength on amorphous carbon during chemical mechanical planarization. Diam. Relat. Mater..

[20] Alghdeir M., Mayya K., Dib M. (2020). Nanosilica Composite for Greenhouse Application. Composite and Nanocomposite Materials-From Knowledge to Industrial Applications.

[21] Gaabour L.H. (2019). Influence of silica nanoparticles incorporated with chitosan/polyacrylamide polymer nanocomposites. J. Mater. Res. Technol..

[22] Bokobza L. (2022). Infrared Linear Dichroism for the Analysis of Molecular Orientation in Polymers and in Polymer Composites. Polymers.

[23] An Z., Wang H., Zhu C., Cao H., Xue J. (2019). Synthesis and formation mechanism of porous silicon carbide stacked by nanoparticles from precipitated silica/glucose composites. J. Mater. Sci..

[24] Phothisarattana D., Harnkarnsujarit N. (2022). Characterizations of Cassava Starch and Poly (butylene adipate-co-terephthalate) Blown Film with Silicon Dioxide Nanocomposites. Int. J. Food Sci. Technol..

[25] Liu X., Zou L., Chang B., Shi H., Yang Q., Cheng K., Li T., Schneider K., Heinrich G., Liu C. (2021). Strain dependent crystallization of isotactic polypropylene during solid-state stretching. Polym. Test..

[26] Ahmed W., Siraj S., Al-Marzouqi A.H. (2021). Comprehensive characterization of polymeric composites reinforced with silica microparticles using leftover materials of fused filament fabrication 3D printing. Polymers.

[27] Botta L., Teresi R., Titone V., Salvaggio G., La Mantia F.P., Lopresti F. (2021). Use of biochar as filler for biocomposite blown films: Structure-processing-properties relationships. Polymers.

[28] Chavan C., Bhajantri R.F., Cyriac V., Bulla S., Ravikumar H., Raghavendra M., Sakthipandi K. (2022). Exploration of free volume behavior and ionic conductivity of PVA: x (x = 0, Y_2_O_3_, ZrO_2_, YSZ) ion-oxide conducting polymer ceramic composites. J. Non Cryst. Solids.

[29] Lin B., Li C., Chen F., Liu C. (2021). Continuous Blown Film Preparation of High Starch Content Composite Films with High Ultraviolet Aging Resistance and Excellent Mechanical Properties. Polymers.

[30] Muñoz P., de Oliveira C., Amurin L., Rodriguez C., Nagaoka D., Tavares M., Domingues S., Andrade R., Fechine G. (2018). Novel improvement in processing of polymer nanocomposite based on 2D materials as fillers. Express Polym. Lett..

[31] Gigante V., Aliotta L., Coltelli M.B., Cinelli P., Botta L., La Mantia F.P., Lazzeri A. (2020). Fracture behavior and mechanical, thermal, and rheological properties of biodegradable films extruded by flat die and calender. J. Polym. Sci..

[32] Wang X., Pan H., Jia S., Wang Z., Tian H., Han L., Zhang H. (2022). In-situ reaction compatibilization modification of poly (butylene succinate-co-terephthalate)/polylactide acid blend films by multifunctional epoxy compound. Int. J. Biol. Macromol..

[33] Cho S.H. (2022). Role of Tie Molecules in Ductility and Chain Deformation of Polyethylene. Ph.D. Thesis.

[34] Karami S., Ahmadi Z., Nazockdast H., Rabolt J.F., Noda I., Chase B.D. (2018). The effect of well-dispersed nanoclay on isothermal and non-isothermal crystallization kinetics of PHB/LDPE blends. Mater. Res. Express.

[35] Li T., Sun H., Lei F., Li D., Leng J., Chen L., Huang Y., Sun D. (2019). High performance linear low density polyethylene nanocomposites reinforced by two-dimensional layered nanomaterials. Polymer.

[36] Wang Z.-Q., Zhang Y.-F., Li Y., Zhong J.-R. (2022). Effect of sodium lignosulfonate/nano calcium carbonate composite filler on properties of isotactic polypropylene. Polym. Bull..

[37] Troisi E., van Drongelen M., Caelers H., Portale G., Peters G. (2016). Structure evolution during film blowing: An experimental study using in-situ small angle X-ray scattering. Eur. Polym. J..

